# Urothelial Malignancy After Normal Hematuria Clinic Investigations: Does Non-visible Hematuria Need Re-investigation?

**DOI:** 10.5152/tud.2024.23025

**Published:** 2024-03-01

**Authors:** Alice Thompson, Bev James, Rotimi David, Mohamed Youseff, Nicholas Gill, Matthew Jefferies, Pradeep Bose, Gokul Kanda Swamy

**Affiliations:** 1 University Hospital of Wales, Urology, Cardiff, UK; 2 Swansea Bay University Health Board, Port Talbot, Neath Port Talbot, UK; 3 Salisbury District Hospital, Salisbury, Wiltshire, UK

**Keywords:** Carcinoma, transitional cell, hematuria, urologic neoplasms, urinary bladder neoplasms, referral and consultation

## Abstract

**Objective:**

Hematuria is the most common referral to Urology. Most initial evaluations are normal; however there are few medium- to long-term studies about these patients after they are discharged.

**Methods:**

This study was a retrospective observational case–control study. Patients with normal initial investigations in our hematuria clinic (HC) over a 2-year period in 2012-2013 were included. We reviewed the electronic records of patients choosing January 1, 2021, as our reference date providing a median follow-up of 99 months. The primary aim of this study was to assess the missed urothelial malignancy (UM) rate in this cohort and also the UM rate in those re-referred to the HC.

**Results:**

The study included 573 patients of whom 24.6% (141/573) were re-referred to urology during the study period. The overall missed UM cancer rate was 0.5% and 0.2% died as a result in this follow-up period. The UM cancer rate in those re-referred was 4.3% and of those re-referred with visible hematuria (VH) the UM cancer rate was 5.7%. No patients re-referred with non-visible VH (NVH) were diagnosed with UM. The only urological death during this time was due to UM.

**Conclusion:**

All urological malignancy and mortality remain very low even at medium- to long-term follow-up after an initial normal HC investigation. In this study, no patients with recurrent NVH developed UM; therefore, recurrent NVH is unlikely to need reinvestigation. The risk of UM in those re-referred with VH is low but more substantial and warrants reinvestigation, which should include computed tomography urogram imaging.

**Main Points**

- The risk of a urological cancer, especially urothelial malignancy, after normal hematuria clinic investigations is very low.
- All visible hematuria representations should be re-investigated, including a computed tomography urogram taking recent investigations into consideration.
- Non-visible hematuria reinvestigation did not identify new urothelial cancers.
- There is no need to re-test for asymptomatic non-visible hematuria after normal hematuria clinic investigations.

## Introduction

Hematuria is the most common reason for referral to Urology.^1-3^ The incidence of urinary tract malignancy, comprising of urothelial malignancy (UM) (bladder and upper tract urothelial cancers) and renal cell cancers (RCC), in patients who present with visible hematuria (VH) is between 18.9-25.2%, and with non-visible hematuria (NVH) is lower at 4.8%-5.9%.^4,5^ A large proportion of patients, therefore, have normal investigations or a benign cause for their hematuria identified.

Recurrent VH and NVH commonly result in re-referral to Urology; however there are no agreed guidelines regarding their management. The American Urological Association (AUA) recommends, based on expert opinion and very low-level evidence, that for those with negative investigations, to consider re-testing at 1 year for patients with NVH or if a patient has further VH.^6^ Conversely, the US Preventive Services Task Force (USPSTF) recommends against routine screening of NVH.^7^ It is also known that hematuria is likely to persist in the majority of patients who are evaluated.^8^

The primary aim of this study is to assess the missed UM malignancy rate in this cohort and the UM malignancy rate in those re-referred to the hematuria clinic (HC) and risk factors associated with these. The secondary aims were to assess UM mortality rates, all urological malignancy and mortality rates, and re-referral patterns.

## Material and Methods

This study was a retrospective observational case–control study that included all patients above 18 years old, who had normal investigations in the HC at our health board in the UK, over a 2-year period between, and including, January 2012 and December 2013. (case = UM, control = no UM).

The primary outcome was the missed UM malignancy rate in this cohort and the UM malignancy rate in those re-referred to the HC. Factors investigated for association with the primary outcome were initial and subsequent hematuria type, findings on initial flexible cystoscopy (FC) and imaging; including ultrasound findings and upper-tract imaging modalities.

Secondary outcomes were UM mortality rate, rate of re-referral to urology, re-referral to the HC, and the rate of all urological malignancy (bladder, upper tract urothelial cancer, renal cancer, prostate, penile, and testis cancer) and all-cause mortality with cause of death reviewed in all patients in the cohort. Outcomes were recorded by accessing electronic patient notes on the regional electronic patient records. The outcomes were assessed as of January 31, 2021, which gave a median follow-up of 8.25 years (range 7.08-9.08 years).

Our center evaluates patients with hematuria in a one-stop HC. It includes FC and ultrasound scan (USS) of the urinary tract at the same appointment. Additional upper tract imaging such as intra venous urograms (IVU) (more common during the study time in 2012-2013) or computed tomography urograms (CTUs) findings were recorded.

The study was initially registered as an audit which allowed the review of patient data. This study was approved by Ethics Committee of Swansea Bay University Health Board (Approval Number: Urlgy/CA/2022-23/05.; Date: May 23, 2022). We referred to the Research Authority Decision Tool, and this project does not constitute research requiring REC/HRA. Informed consent was not required in this retrospective observational study. 

## Results

The total number of patients in the study was 573, which included 329/573 (57.4%) males and 244/573 (42.6%) females, with a median age of 62 years (range of 19-91 years). The ratio of VH and NVH was 2.74 : 1 (420 : 153). Sixty-six percent (378/573) of these patients had additional upper tract imaging at their initial investigation in the form of IVU (350), CTU (51) or both (23), and by the nature of inclusion criteria in this study, had shown no evidence of UM (Table 1.). At this point, 96.7% (554) were discharged and the remaining 19/573 (3.3%) were followed up for reasons including raised PSA (prostate specific antigen), lower urinary tract symptoms, and urolithiasis.

**Figure 1.**
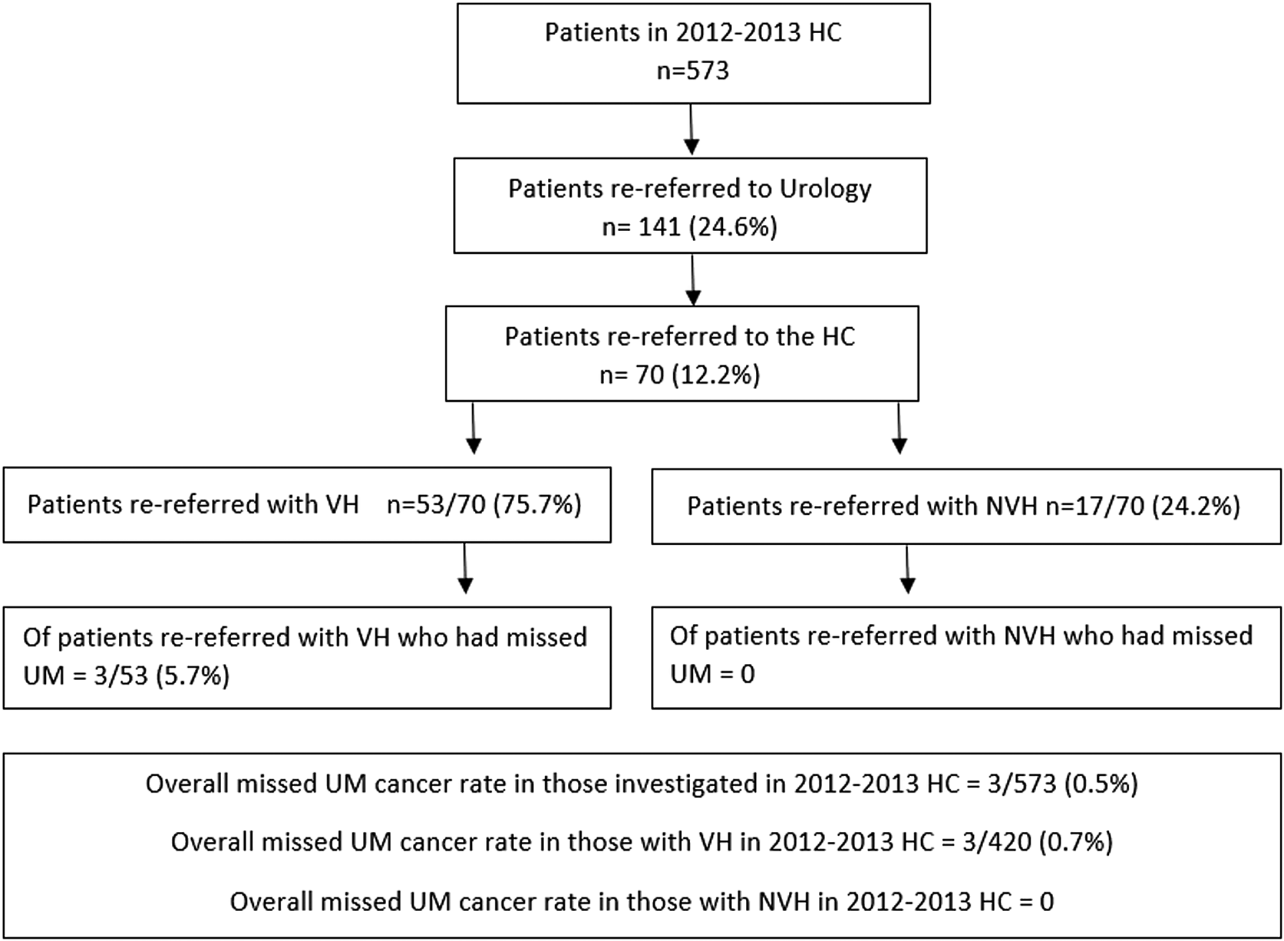
Consort diagram. HC, hematuria clinic; NVH, non-visible hematuria; UM, urothelial malignancy; VH, visible hematuria.

Over the follow-up period, 141/573 (24.6%) patients were re-referred to urology with a male to female ratio of 2 : 1. About half of them, 70/573 (12.2%) were referred for recurrent hematuria back to the HC, with 75.7% (53/70) being referred with VH. The overall missed cancer rate was 0.5% as 3 of the 573 patients developed an UM over this time, and 0.2% (1) died as a result in the follow up period. The UM cancer rate in those re-referred was 4.3% (3/70) and of those re-referred with VH the UM cancer rate was 5.7% (3/53). These outcomes are summarized in Figure 1 and Table 2.

Prostate cancer (PCa) was diagnosed in 1.7% (10/573) of all patients, and 3.8% (2/53) were diagnosed because of recurrent VH. Penile intraepithelial neoplasia was diagnosed in 0.2% (1) but not via the HC. No RCCs or other malignancies were diagnosed. One patient with recurrent VH had Bosniak 2F cyst, and 3 had urolithiasis.

The 3 patients with UM had VH both at initial presentation and at re-presentation. Time to diagnosis from initial HC evaluation was 12, 27, and 61 months. Apart from FC and USS, all 3 had additional upper tract investigation at initial presentation. The details of the presentations, evaluations, treatment, follow-up, and outcomes are all summarized in Table 3. Patient A presented at 24 months with VH and had a FC and USS. He did not have additional upper tract imaging at this point but was then diagnosed with metastatic disease when he presented again after another 3 years.

Totally, 105 patients died over the follow-up period but only 1 death was due to UM. A review of regional electronic patient records included cause of death in all patients and no other urological pathology was evident as the cause for any further deaths.

Since all the UMs diagnosed were in patients presenting at 61 months or earlier, an additional interim analysis was done at the 5-year mark. Half of the re-referrals were due to recurrent hematuria both at 5 and 7 years. There was a similar re-referral rate in subsequent years (approximately 3.5%/year), but the risk of additional UM after 5 years was nil in this cohort.

## Discussion

Despite normal initial hematuria investigations, many patients will be re-referred back to secondary care for further evaluation of recurrent or persistent symptoms. In general, most urologists consider every re-referral for hematuria on its merit irrespective of the previous evaluations as there is limited evidence or guidelines to suggest otherwise.

There are some studies that have investigated the outcomes of patients with normal HC investigations and have shown very low rates (<1-2%) of UMs when re-referred or re-evaluated.^9-15^ Our work adds to the literature as these studies are either short term (2-4 years), low volume, published in abstract form only, or limited to the asymptomatic NVH population, whereas our study is the first comprehensive review of a mixed cohort of patients (not limited to asymptomatic NVH) and review of many additional factors. Furthermore, our study uniquely provides data on both the missed UM cancer rate and the UM rate in re-referrals. The prospective IDENTIFY study reports only the UM rate in re-referrals at 118/1053 (11.2%). And the study by Edwards et al,^14^ reports only the 1.7% missed UM rate, which is similar to the 0.5% in our study.

This data suggests that re-investigating NVH for malignancy is mostly ineffective. Alternative diagnosis such as urolithiasis can be diagnosed but would have associated symptoms. Avoiding HC in these patients would reduce costs and anxiety. It provides evidence against AUA recommendation and in support of USPSTF, to avoid screening for recurrent NVH.^6,7^ Referral to HC is likely therefore not required for recurrent or persistent NVH, unless there are any other symptoms or risk factors of concern. The IDENTIFY risk calculator includes previous benign investigations in its algorithm and can be used to assess who is at high risk when re-referred with hematuria.^4^

All the UMs that were subsequently diagnosed in this study were detected following episodes of VH, which is consistent with recommendations from the literature that recurrent VH should always be re-investigated.^6^ Two of the 3 patients who were subsequently diagnosed with UC were treated with radical treatment and died of competing causes with no evidence of UC recurrence, highlighting the better outcome with early detection and treatment.

It is important to bear in mind that asymptomatic NVH seems to pose a higher risk of developing non-urological adverse events like hypertension, proteinuria, CKD (chronic kidney disease) and even biopsy-proven nephropathies, rather than urological causes. The degree of proteinuria, baseline glomerular filtration rate, and hyperuricemia can predict this.^16^ Presence of proteinuria and NVH suggests intrinsic renal disease and should be referred to renal physicians, and re-testing should be done with a view to detecting nephrological disease rather than urological.

Our study has shown that the chances of any urological malignancy are low at 2.4% at a median of 99 months follow-up, which mostly due to PCa. The risk of UM was very low at 0.5% over the same period. Furthermore, the risk of advanced cancer is very rare at 0.2%.

Hematuria is the most common referral to the urology department^1,2,3^ and the assessments put a high demand on the service and personnel as well as patients who may be subject to health anxiety as well as the invasive tests. Data like this should help counsel patients appropriately and allocate resources fittingly and not burden the service by investigating patients with a very low risk of malignancy.

There are certain limitations to this study. It is a single-center retrospective study. Given the number of patients seen in HC across NHS, it is not a large sample size, but is one of the largest among the studies available in literature so far and far more comprehensive.

Although all mortality is recorded on the electronic patient record for all patients living in our region, it would have been impossible to tell if patients had “moved away” and any mortality or malignancy diagnosed outside the catchment of our regional electronic recording would not have been identified. As patients were not recalled and reinvestigated, there is the risk of asymptomatic patients having UM at the time of this study.

Furthermore, referral patterns and guidelines on initial evaluations have changed since this study population was evaluated, with CTUs replacing IVUs, and changes in NVH evaluations criteria, but none of this would impact the inferences of this study. Since this study focuses on follow-up, it was imperative to have an adequate interval to have meaningful inferences, which qualifies the patient cohort time period.

Although the chance of re-referral to urology increases with time, mostly due to the recurrence of hematuria, the risk of missed UM and subsequent UM mortality remains extremely low after the initial normal HC investigations over a median follow-up of 7 years, and patients can be reassured with these results. Furthermore, the risk of detecting any urological malignancy is also very low at medium- to long-term follow-up.

This study showed no missed cancer in those re-referred with NVH; hence, once evaluated, NVH patients are unlikely to need re-investigation. Therefore, there is no need to re-test asymptomatic patients for NVH with the view to re-referral or re-evaluate for malignancy. There is a small but significant risk of UM in patients with recurrent VH, and this should be re-investigated in the HC and with a CTU, taking recent investigations into consideration.

Until long-term prospective, multicenter, risk factor-based data are available, these data could be potentially used to allay any patient and clinician anxiety.

## Figures and Tables

**Table 1.** Summary of Results from Initial Hematuria Clinic Cohort

Initial HC Patients with Normal Investigation	
Total patients (n)	573
Average age (range)	62 (19-91)
Male to female	329/573 male (57.4%); 244/573 female (42.6%)
VH vs. NVH	420/573 VH (73.3%) vs. 153/573 NVH (26.7%)
Additional upper tract investigation	378 (66.0%) 350/573 IVU (61%); 51/573 CTU (9%); 23/573 Both (4%)

CTU, computed tomography urogram; IVU, intravenous urogram; VH, visible hematuria; NVH, non-visible hematuria.

**Table 2.** Summary of Results for Patients Re-referred to the Hematuria Clinic Including Missed Urothelial Malignancy in Initial Cohort, Rate of Urothelial Malignancy in Those Re-Referred, and Mortality due to Missed Urothelial Malignancy in Initial Cohort

Outcomes of Patients Re-referred to the Hematuria Clinic	
Total re-referred patients (n)	141 (24.6%)
Re-referred to HC	70 (12.2%)
VH vs. NVH	53/70 (75.7%) vs. 17/70 (24.3%)
Additional upper tract investigation	37/70 (52.8%)
Missed UM (in initial cohort) Rate of UM in those re-referred to HC Rate of UM in those re-referred with VH Rate of UM in those re-referred with NVH	3/573 (0.5%) 3/70 (4.3%) 3/53 (5.7%) 0
UM rate in patients with recurrent VH Prostate cancer diagnosed due to VH	3/53 (5.6%) 2/53 (3.7%)
Mortality due to missed UM in initial cohort	1/573 (0.2%)

HC, hematuria clinic; VH, visible hematuria; NVH, non-visible hematuria; UM, urothelial malignancy.

**Table 3.** Urinary Tract Malignancy After Normal Hematuria Clinic Investigations

	Malignancy Type at Re-Rreferral	Initial HC Presentation and Investigations (Month, Year)	Months Until Re-investigation (Reason)	Treatment	LOT Until Death After Initial HC (Cause)
Patient A Male	UT-UC	VH FC, USS, CTU (December 2012)	42 months VH (Normal FC and USS) 61 months Infected obstructed kidney +VH	Palliative	65 months (metastatic UT-UCC)
Patient B Male	UT-UC	VH FC, USS, IVU (April 2012)	12 months (VH)	Radical nephro-ureterectomy	68 months (LRTI) (CT 2018 - no recurrence)
Patient C Male	MIBC	VH FC, USS, IVU	27 months (VH)	Radical radiotherapy	Metastatic lung cancer

CTU, computed tomography urogram; FC, flexible cystoscopy; IVU, intravenous urogram; LOT, length of time; LRTI, lower respiratory tract infection; MIBC, muscle invasive bladder cancer; USS, ultrasound kidney; UT-UC, upper tract urothelial cell carcinoma.
